# Beyond dialysis decisions: a qualitative exploration of decision-making among culturally and linguistically diverse adults with chronic kidney disease on haemodialysis

**DOI:** 10.1186/s12882-018-1131-y

**Published:** 2018-11-27

**Authors:** Danielle Marie Muscat, Roshana Kanagaratnam, Heather L. Shepherd, Kamal Sud, Kirsten McCaffery, Angela Webster

**Affiliations:** 1 The University of Sydney, Faculty of Medicine and Health, School of Public Health, Sydney Health Literacy Lab, Sydney, NSW Australia; 2 The University of Sydney, Faculty of Medicine and Health, School of Public Health, Sydney, NSW Australia; 3 The University of Sydney, Faculty of Science, School of Psychology, Centre for Medical Psychology and Evidence-based Decision-making (CeMPED), Sydney, NSW Australia; 4 The University of Sydney, Faculty of Medicine and Health, Nepean Clinical School, Sydney, NSW Australia; 5 Department of Renal Medicine, Nepean Hospital, Nepean Blue Mountains Local Health District, Sydney, NSW Australia; 6 Department of Renal Medicine and Transplantation, Westmead Hospital, Western Sydney Local Health District, Sydney, NSW Australia; 7 The University of Sydney, Faculty of Medicine and Health, School of Public Health, Wiser Healthcare, Sydney, NSW Australia

**Keywords:** Shared decision-making, Chronic kidney disease (CKD), culturally and linguistically diverse (CALD) patients, haemodialysis, Health literacy, Decision making

## Abstract

**Background:**

To date, limited research has been dedicated to exploring the experience of decision-making for chronic kidney disease (CKD) patients who have initiated dialysis and have to make decisions in the context of managing multiple illnesses. Evidence about the experience of decision-making for minority or disadvantaged groups living with CKD (e.g. culturally and linguistically diverse adults; those with lower health literacy or cognitive impairment) is also lacking. This study aimed to explore the experience of healthcare decision-making among culturally and linguistically diverse adults receiving in-centre haemodialysis for advanced CKD.

**Methods:**

Semi-structured interviews with English or Arabic-speaking adults recruited from four large haemodialysis units in Greater Western Sydney, Australia using stratified, purposive sampling. Interviews were audio-recorded, transcribed verbatim, and analysed using the Framework method.

**Results:**

Interviews were conducted with 35 participants from a range of cultural backgrounds (26 English-language; 9 Arabic-language). One quarter had limited health literacy as assessed by the Single Item Literacy Screener. Four major themes were identified from the data, highlighting that participants had limited awareness of decision-points throughout the CKD trajectory (other than the decision to initiate dialysis), expressed passivity regarding their involvement in healthcare decisions, and reported inconsistent information provision within and across dialysis units. There was diversity within cultural and linguistic groups in terms of preferences and beliefs regarding religiosity, decision-making and internalised prototypical cultural values.

**Conclusion:**

Without sustained effort, adults living with CKD may be uninformed about decision points throughout the CKD trajectory and/or unengaged in the process of making decisions. While culture may be an important component of people’s lives, cultural assumptions may oversimplify the diverse individual differences that exist within cultural groups.

**Electronic supplementary material:**

The online version of this article (10.1186/s12882-018-1131-y) contains supplementary material, which is available to authorized users.

## Background

Chronic kidney disease (CKD) affects up to 10% of the Australian population, with approximately 110 per million population commencing treatment for end-stage kidney disease each year [1]. Over 17 years ago, the Renal Physicians Association and the American Society of Nephrology recommended a shared approach to decision-making for all patients with end-stage kidney disease [2], which is supported by evidence that shared decision-making can improve patient outcomes [3]. Despite shared decision-making emerging as a pillar of national and international quality standards and policies [4], evidence suggests that adults with CKD have limited involvement in treatment decision-making [5, 6]. In addition to transplant and renal replacement therapy decisions (e.g. haemodialysis vs peritoneal dialysis), there are a number of other decisions made throughout the CKD trajectory including those related to lifestyle and diet, medication, long-term dialysis or transplantation, and advance care planning [7]. CKD is also likely to occur alongside multiple comorbid conditions including hypertension, diabetes and cardiovascular disease, where there are multiple test and treatment choices. However, to date, the literature has focused on decision-making about dialysis or transplant options with limited research dedicated to exploring the experience of decision-making for CKD patients who have initiated dialysis and have to make subsequent decisions in the context of managing multiple illnesses.

Evidence about the experience of decision-making for minority or disadvantaged groups living with CKD (e.g. culturally and linguistically diverse adults; those with lower health literacy or cognitive impairment) is also lacking. This represents a significant gap in the literature, given that ethnic minorities in developed countries bear a disproportionate burden of CKD and have worse outcomes [8], and approximately 27% of dialysis patients have limited health literacy [9] (although this proportion has been found to be significantly higher in some studies [10]).

The aim of this study was to explore the experience of decision-making among culturally and linguistically diverse adults currently receiving in-centre haemodialysis for CKD. This work forms part of a larger program of research to engage those with lower health literacy and from culturally and linguistically diverse backgrounds in their chronic care context.

## Methods

We conducted semi-structured interviews with culturally and linguistically diverse adults who were receiving in-centre haemodialysis for advanced CKD between January and October 2017. English interviews were facilitated by R.K. (B.Med/MD candidate) and Arabic interviews were facilitated by N.M (B.Med.Sci, MIPH) both of whom were research assistants trained in qualitative methods and had no previous contact with the participants. Ethical approval was granted by Nepean Blue Mountains Local Health District Human Research Ethics Committee.

### Participant selection and setting

Participants were recruited from four large haemodialysis units in Greater Western Sydney, Australia. In 2016, the population of Greater Western Sydney was 2,232,661, with 38% of the population born overseas and 42% of people speaking a language other than English at home (most commonly Arabic [6.7%]) [11]. Prior to recruitment, we analysed demographic data for participating haemodialysis units. Patient lists confirmed that the two dominant languages spoken during consultations were English and Arabic. Of patients who spoke English during their consultations, the most common regions of birth were in the Pacific Islands (Polynesia) and the Indian subcontinent.

Participants were selected using a stratified, purposive sampling method [12] to represent the dominant cultural and language groups in Greater Western Sydney. We decided a priori to obtain a sample of > 30 to provide sufficient information power using an established model [13]. Participants were eligible to participate if they had sufficient English or Arabic language skills, were older than 18 years, able to give informed consent, and medically fit enough to complete a 30 min interview. The interviewer contacted potential participants at the start of their haemodialysis session, explained the study and invited them to participate. If people agreed to participate, written informed consent was obtained before the interview began.

### Data collection

We developed a preliminary interview topic guide from a review of the literature and discussion with the research team, including three domains: experience of decision-making (renal replacement therapy and other); information and decision-making preferences, and; cultural values. See Additional file 1. At the beginning of each interview we collected demographic information and field notes were made throughout the interviews. Repeat interviews were not conducted and transcripts were not returned to participants.

### Analysis

**Fig. 1:**
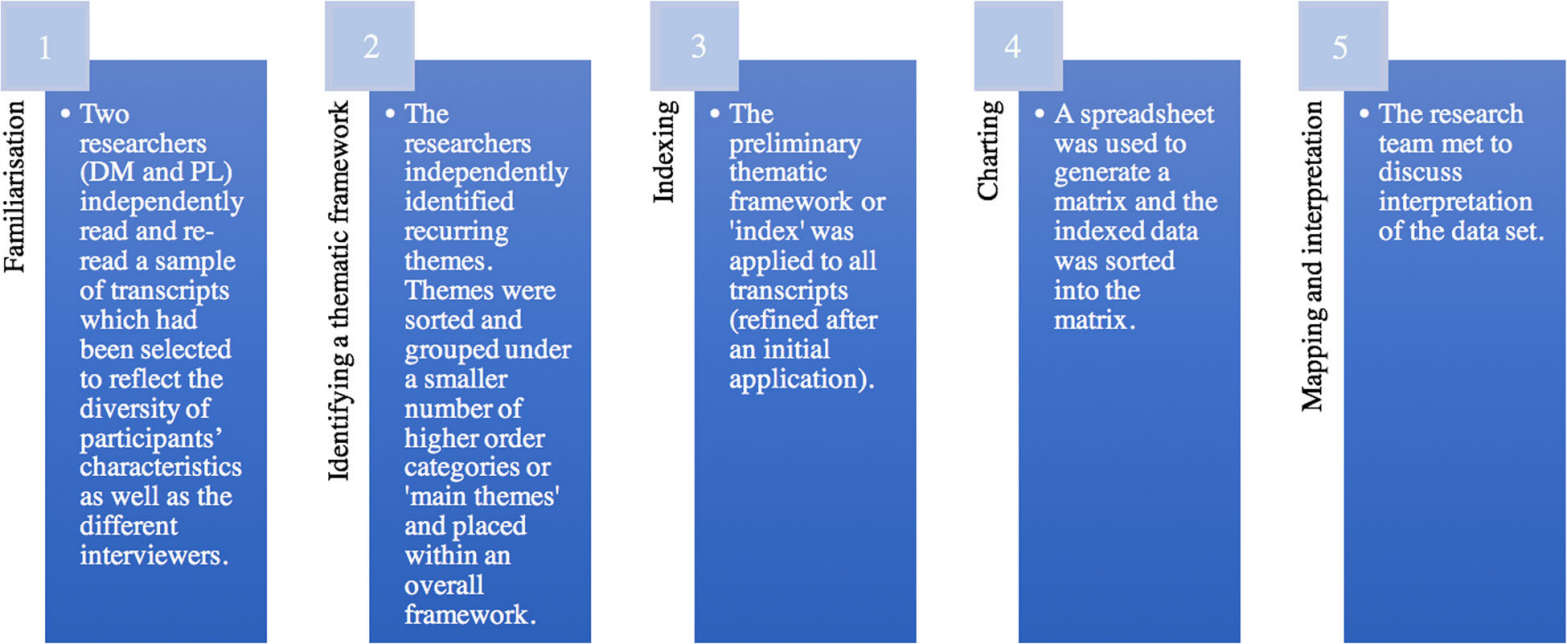
Steps of Framework Analysis

All interviews were digitally audio-recorded and transcribed verbatim by a professional transcription service. For Arabic interviews, transcription was in English. We analysed the transcripts using the Framework approach to thematic analysis, a matrix-based method for ordering and synthesising data [14]. Figure 1.

## Results

Participant characteristics are provided in Table 1. The sample included 35 adults with CKD undergoing haemodialysis. Seven people stated that they were not interested and declined to participate.

**Table 1:** Sample demographics (*N* = 35)

Characteristic	Categories	*N* (%)(total = 35)
Gender	Male	20 (57%)
	Female	15 (43%)
Age (years) *M* = 64.2 SD = 13.6	20–40	3 (9%)
	41–60	8 (23%)
	61–80	20 (57%)
	81–90	4 (11%)
Education level^a^	> 12 years of school completed	16 (50%)
	9–12 years of school completed	7 (22%)
	< 9 years of school completed	8 (25%)
	No formal schooling	1 (3%)
Highest qualifications^b^	Bachelor or higher degree	13 (39%)
	Diploma	1 (3%)
	None	19 (58%)
Place of birth	Australia or New Zealand	2 (6%)
	Europe	1 (3%)
	Middle East	10 (29%)
	South Asia	6 (17%)
	South East Asia	6 (17%)
	Polynesia	10 (29%)
Interview language	English	26 (74%)
	Arabic	9 (26%)
Single Item Literacy Screener (SILS) [35]^c^	1–2 (never or rarely needs help)3–5 (sometimes, often or always needs help; increased likelihood of low health literacy [36])	26 (76%) 8 (24%)

^a^ *Data missing for three participants*

^b^ *Data missing in two participants*

^c^ *The SILS asks, “How often do you need to have someone help you when you read instructions, pamphlets, or other written material from your doctor or pharmacy?”; Data missing for one participant*

Our analysis identified four major themes (with 8 sub-themes) from the data: patient-professional communication; decisional awareness and decision-making; the role of culture, language and religion; family: a cross-cutting theme. Preliminary analysis of early interviews and thematic consistency among interviews conducted across the four haemodialysis units suggested saturation of key themes. Participant quotes are followed by an identification number (PID), gender, age category, country of birth and the language in which the interview was conducted (English or Arabic). Participants with the same number at the beginning of their PID were from the same dialysis unit.

### Patient-professional communication

#### Trust and power-distance

Most participants reported that they felt comfortable talking with members of the healthcare team involved in the treatment and management of their advanced CKD.

“*Oh, I feel comfortable with every doctor. All my doctors I feel comfortable and my specialists. I feel comfortable.*” (PID 1.03, Female, 41–60 years, Samoa, English).

For more than half of all participants, responses reflected a sense of trust in both providers’ integrity and in their competence as healthcare professionals. For example, integrity-based trust was reflected in participants’ assertions that their providers were motivated by concern for the patient and would altruistically act in the patient’s best medical interests.

*“I realised that the doctor want the better outcome for me…they will never give you medication that could cause harm to your health.”* (PID 4.02, Male, 61–80 years, Fiji, English).

Participants also often commented that they perceived their healthcare team to have vast knowledge of their specialty (e.g. “*my doctor knows everything”* (PID 3.07, Female, 61–80 years, Tonga, English)) and expressed belief in their competence as medical professionals. For some participants, competency-based trust was defined in relative terms by assessing the skill differences between healthcare professionals they had encountered in Australia and those from other countries.

*“I trust that the doctors over here more than in the Island. I think it’s because...um...I don’t know if they know what they are doing [laughter].”* (PID 1.04, Female, 41–60 years, Cook Islands, English).

Participants almost always positioned healthcare professionals’ knowledge as superior to their own, reflecting high power-distance in this setting. This appeared to inform their decision-making; many expressed trust in doctors to make a ‘correct’ decision on their behalf.

*“I fully trust my doctor…would do whatever my doctor says.”* (PID 3.01, Female, 61–80 years, Philippines, English).

#### Question-asking as a provider behaviour

When asked about their experiences of asking questions of healthcare professionals, some participants positioned question-asking as a healthcare professional behaviour, rather than a tool for patients to extract information. In fact, for one participant, question-asking was equated to “questioning” healthcare professionals.

*“I don’t ask any question. The doctor asks me questions and I answer.”* (PID 1.03, Female, 41–60 years, Samoa, English).

“*Normally I would not question my doctor whatever she prescribes, I will take it. Why would I question the doctor?*” (PID 4.05, Male, 61–80 years, Sri Lanka, English).

For the participants who reported asking questions, the examples they provided suggested that questions were reactive, that is, often asked in response to deteriorating health conditions rather than to actively seek out unprompted information.

*“Only ask her when something wrong with me, that’s it.*” (PID 1.02, Female, 61–80 years, Samoa, English).

One clear exception was the participant below who expressed that he was comfortable asking his healthcare team anything about his health.

*“With my doctors, I ask them anything and I don’t hold anything from them. I also don’t fear to ask them anything if I need to.”* (PID 4.14, 61–80 years, Male, Egypt, Arabic).

### Decisional awareness and decision-making

#### The centrality of the renal replacement therapy (RRT) decision

When asked about decisions that they have had to make regarding their healthcare, few participants referred to anything other than choices about dialysis modality, even when prompted. One exception was a female participant who referred to her decision to “change eating habits” after initiating dialysis. Notably, although many participants alluded to aspects of their treatment or care which would have had a decision point, these were not positioned as decisions in which they were actively involved. For example, many participants referred to medications that they were taking throughout the interviews, but did not discuss medication initiation or cessation as a decision point.

#### RRT decision-making and dialysis initiation: A tale of variability

##### Preferences for information

With regards to beginning dialysis, participants’ responses reflected an expectation to receive information from providers. Most often, information was positioned as important so that patients would know what to expect from the dialysis process; it was not seen as a tool to inform decision-making prior to commencing treatment.

*“They should tell you clearly what to expect*. *Patients should be given some videos or link in order to prepare them. Such information could take away a lot of pressure on the kidney patient.”* (PID 4.16, 61–80 years, Male, Lebanon, Arabic).

##### Information provision

Both within and across dialysis units, participants described varied experiences of information provision related to dialysis decision-making, from receiving detailed information about dialysis choice via attendance at dialysis information days and counselling sessions, to receiving no information at all.

*“Doctors provided all the health-related information including the options available, side effects, pros and cons of each option and even sent me to this education, um, program on dialysis in the hospital.”* (PID 4.03, Female, 41–60 years, India, English).

*“Not the doctor...Actually the counsellor from that hospital, she gave me a lot of information about dialysis. I think I had two or three sessions with her. She gave me some – some books and some research from dialysis.”* (PID 4.07, Male, 20–40 years, Pakistan, English).

*“No one told me anything. There’s no lecture at all…I got all the information from internet…”* (PID 2.01, Male, 81–90 years, Malaysia, English).

##### Preferences for decision-making

**Fig. 2:**
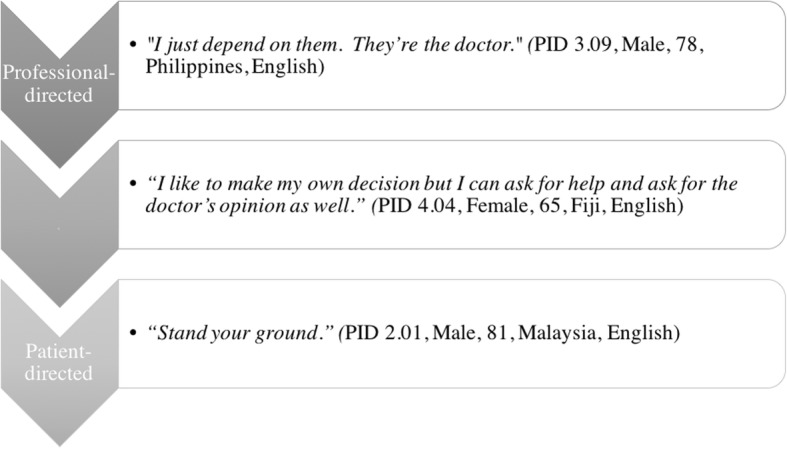
Preference for making the dialysis modality decision

Preferences for being involved in decision-making were also varied. Respondents expressed preferences ranging from physician-directed to patient-directed styles for making the final decision about renal replacement therapy. See Fig. 2.

##### The decision-making experience

In a similar way to preferences for decision-making being varied, the reported experience of participants in decision-making was very different. While some felt that they were involved in decisions about starting dialysis, others did not. Table 2 provides a list of perceived barriers to involvement in dialysis decision-making expressed by participants throughout the interviews.

**Table 2:** Perceived barriers to involvement in dialysis decision-making

Perceived barrier	Exemplary quote
Clinical directive/physiological contraindications	*“First I was put on peritoneal dialysis, but, but PD catheter didn’t work…it was a disaster so now I am on haemodialysis.”* (PID 3.01, Female, 61–80 years, Philippines, English)
Insufficient information provision	*“No one supported me. I would have felt better supported from the medical staff if I had enough information.”* (PID 4.11, 20–40 years, Male, Lebanon, Arabic)
Paternalistic ideologies	*“I didn’t have much choice of which one I can take. I had to take the one which my doctor recommended…There are occasions where I said ‘I didn’t want’ and all that, but then at the end of the day, they have their way [laughter].”* (PID 3.05, Male, 61–80 years, India, English)
Time pressures	*“…did not give me enough time to decide over the options. It went very fast from first information session and starting dialysis.”* (PID 4.06, Female, 41–60 years, Tonga, English)
Lack of awareness of different options	*“All they do is talk about among themselves, so I was in the dark what, what is to be done.”* (PID 2.01, Male, 81–90 years, Malaysia, English)

### The role of culture, language and religion

#### Religion

For some, religion and faith were unimportant, however the majority of participants (60%) indicated that faith was central to their lives and cultural identity, and informed their understanding of their illness and prognosis. Religious leaders and groups offered practical and spiritual support to participants, and religious beliefs were expressed as a way of coping with illness.

*“I believe. Well that’s my belief. Because I always ask God, you know, to heal the pain…and it’s gone…If I’m in pain I cry out to God to take the pain away and it’s gone.”* (PID 1.04, Female, 41–60 years, Cook Islands, English).

*“I was really sick. And then I went to church. They bless me. All of them are blessing me…That’s why I am still here.”* (PID 1.03, Female, 41–60 years, Samoa, English).

However, no participant reported any direct impact of religion on decision-making and one fifth of participants explicitly expressed that they did not think it necessary or useful to discuss their faith with their healthcare team unless it practically impacted their treatment or care or the doctor had asked directly. One participant expressed this as a clear delineation between “science” and “religion”.

*“Religious [sic] is an important part of my life but it doesn’t disturb my health, with the talking to doctor and things like that. Sometimes doctor asks, “Do you eat meat?” Well, we don’t.”* (PID 1.04, Female, 41–60, Cook Islands, English).

#### Culture, language and communication

When asked about the influence of cultural background on healthcare and healthcare interactions, all but three participants thought that culture did not influence their communication with their healthcare providers. The few who thought that it did stated they could better relate to providers who shared their cultural background and beliefs.

*“It’s easy to talk to the doctor with same subcontinental background as you relate stuff like predisposition of same kind of diseases, the doctor is already aware of.”* (PID 4.08, Male, 41–60 years, India, English).

At times, citizenship/residency status impacted participants’ perception of available treatment options such as eligibility for transplantation.

*“Oh, actually they suggest me to the transplant soon, but the problem is this; because I am from, um, Pakistan and I’m overseas here, so according to the Australian law I’m not eligible to put my name in the transplant list.” (*PID 4.07, Male, 20–40 years, Pakistan, English).

#### Language

Although some participants reported that language was not a barrier to them communicating with their healthcare team or others in English, interviewers noted difficulties in communication throughout the English-language interviews. Participants did acknowledge that understanding their providers and expressing their concerns and questions was much easier in language-concordant consultations.

*“Yeah, definitely – definitely because you can easily describe your [sic] each and everything and they can understand.”* (PID 4.07, Male, 20–40 years, Pakistan, English).

To overcome language barriers, adult children often accompanied patients from diverse linguistic backgrounds to their appointments, especially specialist appointments where there was more often a language discordance between provider and patient.

“*My children are the one with me at every visit since I don’t speak good English.”* (PID 4.05, Male, 61–80 years, Sri Lanka, English).

#### The role of the general practitioner (GP)

Participants often reported that they had a more enduring relationship with their GP compared to their renal specialist, and that their local GP was often from the same cultural, linguistic and/or religious background as they were. In this context, communication was deemed to be easier.

*“I can tell her and discuss anything with my GP simply because she understands me easily.”* (PID 4.13, 61–80 years, Female, Lebanon, Arabic).

In fact, for one patient, the local GP acted as intermediary between the patient and the specialist, translating the information that had been provided in specialist consultations.

*“no language barrier with GP as he can explain everything in Tongan language. But the specialist is not Tongan…So when we go to the GP he would explain about what the specialist would say. And he will then explain…in Tongan why.”* (PID 3.02, Female, 61–80 years, Tonga, English).

In addition to the perceived benefits of the GP speaking the same language as the patient, participants also alluded to aspects of religion and culture. The female participant above, for example, went on to describe how the GP consultation represented a safe space to discuss complementary and alternative medicines from Tonga given the shared cultural background between her and her provider. Similarly, the participant below said he felt comfortable discussing his religious beliefs with the GP who he knew shared his religious affiliation but did not feel comfortable doing so with the renal specialist.

*“…I talk to my GP about religion as my GP is also catholic but I don’t talk about religion to my Kidney doctor. It may be inappropriate to discuss religion, because I don’t know him well, he is Greek.”* (PID 3.06, Male, 61–80 years, Philippines, English).

### Family: A cross-cutting theme

The role of the family was cross-cutting in that family members were often positioned as providing both practical support (e.g. language) and influencing decision-making. Family can also be seen as cultural dimension insofar as it relates to notions of individualism and collectivism. In practical terms, participants reported that their families often drove them to their dialysis sessions and supported their communication with the healthcare team.

*“The family, they like to be involved in case if I miss something, they will explain it to me. My eldest daughter works with doctors and every time when I have an appointment with the doctor she will come and drive me and go in with me for the appointment.”* (PID 4.15, 61–80 years, Male, Lebanon, Arabic).

Families were involved in decision-making to different degrees and in different ways. Table 3 includes examples of where decisions were made *by* family members, *with* family members, and *for* family members.

**Table 3:** Influence of family on decision-making

Influence of family in decision-making	Exemplary quote
Decisions made *by* family	*“...initially my daughter was not happy for me to do the dialysis. The doctor explained to her that I might have a better chance of survival if I do the dialysis than not doing it. Once she was convinced for me to undergo dialysis, then only I went for it.”* (PID 4.05, Male, 61–80, Sri Lanka, English).
Decisions made *with* family	*“My children, the doctor and my wife they convinced me to undergo dialysis. At the beginning, I have refused and I did not want to undergo dialysis but when they told me (my children) that they do not want to lose me then I thought it’s better to do it.”* (PID 4.02, Male, 61–80 years, Fiji, English)
Decisions made *for* family	“*I think about my family. My Kids. So it’s always about my kids. I want to live longer so my little ones I can see them grow up*.” (PID 1.03, Female, 41–60 years, Samoa, English)

## Discussion

This qualitative interview study involving 35 patients with CKD undergoing haemodialysis in Greater Western Sydney, Australia has contributed novel and confirmatory findings related to healthcare decision-making for culturally and linguistically diverse patients. Our results have implications for practice and for the development of shared decision-making interventions.

Our findings demonstrate the significance of the decision to start dialysis for patients. While this may be unsurprising given the tangible impact this decision has on their daily lives, our study has identified its prominence over-and-above all other decisions made throughout the CKD trajectory (e.g. decisions to change diet and lifestyle; medication decisions). This may suggest a lack of awareness of other decision points (low decisional awareness), limited engagement in the process of making these decisions (low decisional engagement [15]), and/or the fact that patients do not consider decisions about diet and lifestyle, medications and advance care planning as decisions at all. Given that a shared approach to making such decisions may result in a greater adherence to the chosen treatment than to treatment decisions made by the healthcare professional alone [16], there is need to broaden the way we communicate about decision-making to enable patients to appreciate the multiple decision points throughout their illness trajectory and the value of actively engaging in decisions about their health. This also offers an important avenue for future research and decision-support tool development in CKD which has, to date, concentrated on dialysis initiation and transplantation choices.

Our work has also highlighted varied levels of involvement in decision-making about dialysis initiation. As we have found, recent literature attests that patients with advance stage kidney disease report varied experiences regarding involvement in dialysis decision-making [5, 17–20], and has shown that decisions about dialysis can be time-pressured and stressful for patients who are often very sick. Clinical directives or physiological contraindications can limit the treatment options available for consideration [21], and paternalistic ideologies about decision-making are not uncommon for CKD patients and healthcare teams [22]. However, our study has also identified variable information provision within and across dialysis units as a barrier to shared decision-making. Perceived variability in information provision about dialysis may reflect the reality that different units offered different information to patients, and/or that different patients within the same unit received different information and access to information services. Alternatively, it may be that some patients were not able to access or understand information available within their renal unit, as a large proportion of information for CKD patients is pitched above the average patient’s literacy level [23] and few resources are available in languages other than English. These findings highlight the need for greater consistency in information provision to support all patients to be involved in decision-making, and the need to support information provision with efforts to improve health literacy skills of patients and reduce the literacy demands of existing resources.

Our findings also show that participants expressed high levels of trust in providers. This can be both facilitative and inhibitory in terms of decision-making. Qualitative studies exploring decision-making within other cultural groups in the context of CKD have found that trusting relationships make it more likely for patients to confide and share personal information with their providers, as well as ask for and receive help [24]. In a study involving Māori participants, trusting relationships were seen as a crucial to maintaining engagement and active participation with clinical services [25]. However, our findings show that question-asking was often positioned as a provider behaviour and participants almost always positioned healthcare professionals’ knowledge as superior to their own. In the context of trusting patient-provider relationships this suggests that too much trust in the healthcare team can create passivity that is an obstacle to shared decision-making [22]. This highlights a need to support patients to place trust in professionals balanced by an awareness of the unique contributions that they bring to decision-making (such as the contribution of knowledge of their personal contexts, values and preferences).

This work has also highlighted the importance and prominence of family in decision-making about dialysis. While previous studies have found that family informs decision-making for specific cultural minority groups (e.g. Māori patients), our purposive sampling of diverse cultural groups extends on this by highlighting similar importance cross-culturally. This supports a more global view of the importance of family in CKD decision-making and suggests that, in this setting, the importance of family and family support may be *transcultural* rather than *cross-cultural* in that it transcends cultural boundaries [26]. Earlier reviews had similarly found that patients across geographical boundaries rely on family members for practical, intellectual and emotional support throughout their illness and were mindful that their choice about dialysis would also affect their families [27]. Together, this lends support to the conceptual framework of triadic decision-making which reinforces the need to involve family members in decision-making and in decision-making interventions [28]. It will also be important to explore the role families play at other decision points throughout the CKD trajectory (in addition to renal replacement therapy) and their role in supporting adherence to decisions made (e.g. maintenance of dietary and lifestyle changes).

Our diverse sample has also highlighted the variation within cultural and linguistic groups in terms of preferences and beliefs regarding religiosity, decision-making and internalized prototypical cultural values. This opposes cultural theories which imply that cultural groups are homogenous [29] and reinforces the importance of acknowledging individual differences both *within and across* cultures [30]. For example, there were not only differences in religious beliefs within our sample, but also differences in the value placed on religion as well as reported differences in preferences for involvement in decision-making (which has been shown elsewhere [31]). Together, these findings reinforce the need to tailor and target communication and decision-making style to the individual rather than rely on cultural assumptions.

However, internalised cultural norms affect us in subconscious or nuanced ways. Hofstede and others [32], analysed 5820 audio-recorded medical consultations and found that cultural differences across four dimensions (individualism/collectivism; uncertainty avoidance; power distance; masculinity/femininity and long-term orientation) affected medical communication in general practice: the greater power distance, for example, was associated with less unexpected information exchange and with more clearly defined roles for patients and providers. Similarly, although religion was not perceived to effect decision-making, for those whom religion played a central role in their lives, recognising this may be important to facilitate a therapeutic relationship between provider and patient. In previous studies, for example, clinicians were considered more trustworthy when they knew and discussed what was valued and important to their patients, such as their spiritual connections to their land and people [25]. It may also play a more central role in other decisions, such as those regarding end-of-life.

### Strengths and limitations

Purposive sampling enabled us to capture experiences of patients from different cultural groups representative of the linguistic and cultural diversity in Australia [33] and those living with advanced stage CKD [34]. The use of bilingual interviewers meant that we were able to include individuals who may otherwise be excluded from research studies. However, this was limited to English and Arabic speakers. Additionally, the task of reflecting on decisions throughout the illness trajectory was inherently difficult and subject to recall biases, and there remained linguistic challenges for culturally and linguistically-diverse patients in participating in English language interviews. Additionally, participants were heterogeneous outside their cultural background (e.g. age, education level) and we did not specifically explore the influence of these other multiple demographic dimensions on decision-making. However, they would have likely influenced disease management and decision-making. Qualitative interview methodologies may not have captured some important cultural influences on decision-making, and so observational studies offer a promising approach to complement this work in CKD settings the future.

### Directions for research and practice

Findings from this study point to specific recommendations about how to improve decision-making in this group of patients. See Table 4.

**Table 4:** Recommendations about how to improve decision making for this group of patients

Key finding	Recommendations to support decision making
Participants demonstrated a high level of trust in healthcare professionals and the perception that professionals’ knowledge was superior to their own.	- Reinforce unique contribution of patients to decision-making (e.g. knowledge of personal contexts, values and preferences). - Acknowledge that there are no right or wrong answers in situations of equipoise [37].
Patient question-asking in healthcare settings appeared limited and reactive.	- Redefine perceptions of a good patient and reassure patients that participation and question-asking will not result in retribution [38]. - Enable patients to ask questions through the provision of question prompt lists [38].
Limited awareness/engagement in decisions other than RRT throughout CKD trajectory.	- Develop decision aids for decisions other than renal replacement therapy. - Reframe patient education materials to reinforce choice and patient engagement in formulating and adhering to treatment goals (e.g. diet and fluid restriction) [37].
Variability in: - Preferences for decision making - Amount of information provided to patients - Decision-making experiences	- Assess preferences for information and decision making. - Centralise resources across institutions, such as through an online repository. - Utilise health information technologies for the delivery of consistent information to patients regardless of institution/location. - Develop translated and culturally-adapted resources which are appropriate for the health literacy level of patients. - Provide training for health professionals (at all stages) to support shared decision-making [39]. - Provide training for patients to build skills and capacity to engage in decision-making [40].
Religion perceived as important to many people, but few thought it concerned healthcare decision-making.	- Clarify and agree on preferences regarding the discussion of religion upon commencement of consultation.
Family played an important role in decision-making.	- Develop and disseminate decision support tools for family members. - Clarify and agree on preferences and roles for family members upon commencement of consultation [41].
Importance and value of GP	- Explore ways to facilitate shared decision-making within integrated care models, such as through training in inter-professionalism [42].

## Conclusions

Implementing shared decision-making into routine practice and targeting and tailoring information to accommodate diverse cultural groups and individual differences is a considerable challenge. The results of this qualitative study reflect the significant need to address this in the CKD context. Without sustained effort, patients may be uninformed about decision points throughout the CKD trajectory, unaware of their rights and potential contribution to decision-making and/or unengaged in the process of making decisions. While culture may be an important component of people’s lives, cultural assumptions may oversimplify the diverse individual differences that exist within cultural groups.

## Additional files

Additional file 1: Qualitative Interview Topic Guide. (DOCX 20 kb)

## Abbreviations

B.Med.Sci: Bachelor of Medical Sciences
B.Med/MD: Bachelor of Medicine
CKD: Chronic kidney disease
GP: General practitioner
MIPH: Masters in International Public Health
PID: Participant Identification
