# Supplementary material for: Beyond dialysis decisions: a qualitative exploration of decision-making among culturally and linguistically diverse adults with chronic kidney disease on haemodialysis
Source: BMC Nephrol. 2018 Nov 27;19:339. doi: 10.1186/s12882-018-1131-y (PMC6258454; doi:10.1186/s12882-018-1131-y)
Supplement: Supplementary file 1 — Qualitative Interview Topic Guide. (DOCX 20 kb) [file 12882_2018_1131_MOESM1_ESM.docx]

**Qualitative Interview Schedule: A qualitative exploration of decision-making among culturally and linguistically-diverse adults with chronic kidney disease on haemodialysis**

1. Introduction
   1. Explanation of study
   2. Obtain informed consent
   3. Demographic information
   4. CKD-specific health information
2. Experience of decision-making throughout the CKD trajectory
   1. Experience of engaging with healthcare professionals
   2. Experience of decision making regarding RRT and dialysis choice
   3. Experience of decision making for decisions other than dialysis initiation
3. Information and decision making preferences
   1. Preferences for information
   2. Preferences for decision making
4. Cultural values and influences
   1. Perceived role of culture regarding engagement with healthcare professionals and decision making
   2. Hofstede’s cultural dimensions: independence/interdependence and power distance
   3. Perceived role of religion regarding decision making
   4. Comparison of healthcare consultations and decision making in home country and Australia
5. Conclusion
   1. Questions and/or additional comments from interviewee
   2. Thank-you and interview end
